# Competency of *Anopheles stephensi *mysorensis strain for *Plasmodium vivax *and the role of inhibitory carbohydrates to block its sporogonic cycle

**DOI:** 10.1186/1475-2875-7-131

**Published:** 2008-07-15

**Authors:** Hamid R Basseri, Soghra Doosti, Kamran Akbarzadeh, Mehdi Nateghpour, Miranda MA Whitten, Hossein Ladoni

**Affiliations:** 1 Department of Medical Entomology, School of Public Health, Tehran University of Medical Science, Iran; 2 School of the Environment and Society, Swansea University, Singleton Park, Swansea, SA2 8PP, UK

## Abstract

**Background:**

Despite the abundance of studies conducted on the role of mosquitoes in malaria transmission, the biology and interaction of *Plasmodium *with its insect host still holds many mysteries. This paper provides the first study to follow the sporogonic cycle of *Plasmodium vivax *in a wild insecticide-resistant mysorensis strain of *Anopheles stephensi*, a major vector of vivax malaria in south-eastern Iran. The study subsequently demonstrates that host-parasite sugar binding interactions are critical to the development of this parasite in the salivary glands of its mosquito host. The identity of the receptors or sugars involved was revealed by a receptor "pre-saturation" strategy in which sugars fed to the mosquitoes inhibited normal host-parasite interactions.

**Methods:**

*Anopheles stephensi *mysorensis mosquitoes were artificially infected with *P. vivax *by feeding on the blood of gametocytaemic volunteers reporting to local malaria clinics in the Sistan-Baluchistan province of south-eastern Iran. In order to determine the inhibitory effect of carbohydrates on sporogonic development, vector mosquitoes were allowed to ingest blood meals containing both gametocytes and added carbohydrates. The carbohydrates tested were GlcNAc, GalNAc, arabinose, fucose, mannose, lactose, glucose and galactose. Sporogonic development was assessed by survival of the parasite at both the oocyst and sporozoite stages.

**Results:**

Oocyst development was observed among nearly 6% of the fed control mosquitoes but the overall number of mosquitoes exhibiting sporozoite invasion of the salivary glands was 47.5% lower than the number supporting oocysts in their midgut. Of the tested carbohydrates, only arabinose and fucose slightly perturbed the development of *P. vivax *oocysts at the basal side of the mosquito midgut, and the remaining sugars caused no reductions in oocyst development. Strikingly however, sporozoites were completely absent from the salivary glands of mosquitoes treated with mannose, GalNAc, and lactose.

**Conclusion:**

The study indicates that *An. stephensi *in southern Iran has the potential to survive long enough to be re-infected and transmit vivax malaria several times, based on the average adult female longevity (about 30 days) and its gonotrophic cycle (2–3 days) during the malaria transmission season. Certain sugar binding interactions are important for the development of *P. vivax *sporozoites, and this information may be instrumental for the development of transmission blocking strategies.

## Background

Malaria remains the most devastating vector-borne disease despite a century of concerted international efforts to control it. Most antimalarial strategies have primarily targeted the infection in humans and the control of mosquitoes with insecticides. These approaches have proven unsuccessful, in part because parasites have developed resistance to antimalarial drugs, and mosquitoes have become resistant to insecticides. The malaria parasite *Plasmodium *takes many forms during its complex life cycle in the vertebrate host and invertebrate vector [1]. Our understanding of the basic biology of vector-parasite interactions during the transit of the parasite through the mosquito is still limited [2]. Upon ingestion, *Plasmodium *gametocytes differentiate into gametes within the midgut lumen, fertilization takes place and the zygote develops into an ookinete, which penetrates the midgut epithelium and forms oocysts on the basal side. When the oocysts mature and subsequently rupture, sporozoites are released into the haemolymph and invade the salivary glands, to be injected into a new host when the infected mosquito feeds again. Diverse mosquito species differ in their ability to transmit different *Plasmodium *parasites, and stage-to-stage specific losses seem to depend on the vector and the parasite species [3]. A certain mosquito species always shows different permissiveness to different *Plasmodium *species. For instance, *Plasmodium berghei *will develop at least 50 oocysts in *Anopheles gambiae*, while *Plasmodium falciparum *rarely exceeds three to four oocysts per midgut in the field and even in laboratory infections [4].

There are several possible new approaches to interrupt the malaria transmission cycle in the mosquito, including mosquito vaccines [2], transmission-blocking vaccines [3] or transmission-blocking antimalarial drugs [5,6]. Understanding the life cycle of medically-important *Plasmodium *species/strain combinations is crucial for the development of vaccines, and the design of an effective transmission-blocking strategy depends on a full molecular understanding of the sporogonic development of the parasite in the mosquito [2]. During sporogony, a parasite's transition from one developmental stage to another is confined to specific tissue compartments within the mosquito. Each compartment contains specific factors that interact with the parasite and influence its development. Potentially the role of carbohydrate receptors represents one of the most important keys to understanding vector/parasite interactions [7]. A variety of sugar linkages are present on the surface of the mosquito midgut epithelium, some of which partially block attachment of malaria ookinetes to the midgut surface *in vitro *[8]. Thus, the mosquito midgut epithelium, similar to the lining of mammalian intestines, is extensively covered with surface carbohydrates that may play a role in pathogen attachment.

Recently evidence for a "protein-carbohydrate recognition strategy" for blocking vector host-pathogen interactions has emerged. Through a combination of conventional and novel approaches, oligosaccharide mimics or antiglycan antibodies are being developed that could help to reduce disease transmission [9]. For example, antiglycan antibodies that are taken up by a mosquito along with a parasite infective blood meal, can mask parasite glycan receptors that are critical for attachment to the midgut [10]. In addition, glycoproteins are potential transmission-blocking vaccine candidates for insect vector-borne diseases and further reinforce the importance of understanding the effects of carbohydrate epitopes in development of vaccines [11]. Anti-carbohydrate antibodies successfully block *Plasmodium gallinaceum *ookinete adhesion to the midgut of *Aedes aegypti*, suggesting that carbohydrate residues are used by the parasite for recognition and binding to the midgut [7]. Furthermore, antibodies to *Anopheles tessellates *midgut glycoproteins that contain GalNAc side chains inhibit the infectivity of the two major human malaria parasites, *P. falciparum *and *Plasmodium vivax *[12].

Three strains of *Anopheles stephensi *are recorded in the south of Iran [13], but *An. stephensi *strain mysorensis is the dominant vector in the Sistan-Baluchistan province of southeastern Iran, where transmission of vivax malaria is particularly high. Annually about 60% of the malaria cases in Iran are reported from this province (during this study, the number of positive cases was 11,305 including 9,245 *P. vivax*, 1,926 *P. falciparum *and 134 mixed infections with both species of *Plasmodium*). The current annual parasite incidence (API) is 7 per 1,000 inhabitants (Center for Disease Control, Ministry of Health, Iran). In Sistan-Baluchistan province, *P. falciparum *is also resistant to anti-malaria drugs [14]. Additionally, the two most important vectors in this region, *An*. *stephensi *and *Anopheles culicifacies*, are both resistant to organochlorine insecticides [15]. Novel transmission-blocking and/or control strategies are urgently needed for this region of Iran. The importance of highly species- and strain-specific differences in host-parasite interactions, and consequent strategy design, cannot be overstated [16].

Therefore, the current investigation adopted a geographically-relevant study to describe the competency of the *An*. *stephensi *mysorensis vector for *P. vivax*, using insects collected from the field and parasites from patients living in the same region. The developmental kinetics of the sporogonic life cycle were investigated in detail to optimize studies on the effect of ingested carbohydrates on the two most vulnerable stages of parasite development: ookinetes and sporozoites. It was found that *An. stephensi *mysorensis is a very competent vector for *P. vivax*, but that sporozoite invasion of the salivary glands can be powerfully blocked by the carbohydrates mannose, GalNAc, and lactose. These findings will assist the development of a specific TBV strategy targeted to this important region of Iran.

## Methods

### Mosquitoes

In order to establish a colony of wild *An. stephensi *strain mysorensis, mosquito larvae were collected from natural breeding paces in Iranshahr district, Sistan-Baluchistan province, Southeastern Iran, and then transferred to the insectary of a local research centre. Larvae were reared in bowls at a density of 300 larvae/500 ml of water. The pupae were transferred to cages made of muslin cloth for harbouring adults. All mosquitoes were maintained at 28 ± 2°C and 70 ± 10% relative humidity. During rearing, the adult mosquitoes were fed on 5% sucrose solution and the females on rabbit blood. The mosquitoes were allowed to regenerate three times in order to boost the population of colonies, and F3 mosquitoes were used for the experiments. For experiments (see below for details), nulliparous female mosquitoes were starved for 12–18 hours before feeding on a blood meal containing *P. vivax *gametocytes.

### Parasites

Gametocytes of *P. vivax *were obtained from resident volunteer malaria patients who attended local clinics in the Iranshahr district, Baluchistan province. Patients who had gametocytes in their blood were chosen for interview and to avoid damaging the parasites, only those patients who had not previously taken any anti-malarial drugs for the current infection, were chosen as donors. Informed consent was obtained from all individuals participating in this study, and ethical approval was obtained from the Ministry of Public Health via the approvals procedure of Tehran University of Medical Sciences. All malaria patients were followed up by local Health Services personnel.

### Sporogonic development

In order to determine exflagellation events, and oocyst and sporozoite development, mosquitoes were sampled at several time-points post-infection. Following infective membrane feeding, unengorged females were discarded and the fed mosquitoes were maintained in standard conditions, as described above, until assessed for sporogonic development. In order to measure the time of exflagellation, mosquitoes were dissected (five fed females each time) at one-minute intervals between one and five minutes post feeding, and then at five-minute intervals until the 30^th ^minute post feeding. To assess ookinete development, dissection of mosquito midguts was performed at regular intervals between 12 and 24 hours post-infection. To assess oocyst and sporozoite development, the midguts and salivary glands of mosquitoes were surveyed from 8 dpi until sporozoites were observed in the salivary glands. All dissected midguts and salivary glands were stained with mercurochrome and Giemsa (Merk Co., Germany) and observed with phase-contrast microscopy (200 and 400 × magnification).

### Carbohydrate inhibition assay

By characterizing *P. vivax *development in *An. stephensi *strain mysorensis, it was then possible to optimize an assay to evaluate *in vivo *carbohydrate inhibition effects on parasite development in the midgut and salivary glands (the two major population bottlenecks for *Plasmodium*). Mosquitoes were membrane-fed with gametocytaemic blood containing 0.2 M carbohydrate (based on a concentration that was optimized previously by Chen and Billingsley [17]). The following carbohydrates were selected for study because their residues had previously been detected on the surface of mosquito salivary glands and midguts [18,19]: N-acetyl-glucosamine (GlcNAc), N-acetyl-galactosamine (GalNAc), arabinose, fucose, mannose, lactose, glucose and galactose. All the mosquitoes used in the sugar-feeding experiments were nulliparous females from the F3 generation cohort. For each experiment, insects were randomly separated into nine groups, with each group containing at least 100 insects. Each group was treated with a different carbohydrate (or no carbohydrate in the control group) and all nine groups were fed using the blood from a single donor. This experiment was then independently repeated a further two times, each time using 9 further groups of mosquitoes feeding on blood from a new donor. Unfed mosquitoes were discarded and the engorged mosquitoes were split into two further groups and maintained until assessed for parasite development in the midgut (at 9–10 days post-infection) or in the salivary glands (15 days post-infection), as described above.

## Results

### Development of sporogony

**Figure 1:**
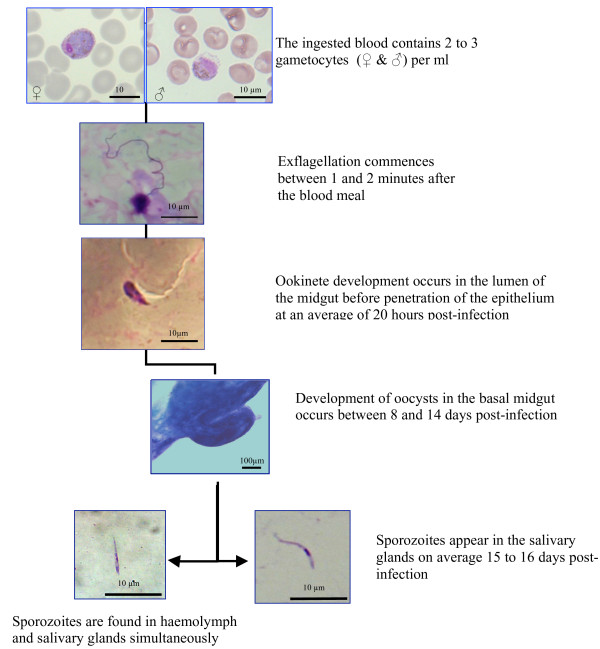
**Sporogonic cycle and timing of the development of *Plasmodium vivax *in the different compartments of *Anopheles stephensi *strain mysorensis**. Each stage of *Plasmodium *development was directly observed with light microscopy immediately after dissection. Giemsa staining.

In this study, the development of *P. vivax *in *An. stephensi *strain mysorensis has been examined. Exflagellation of the parasite started within an average of one to two minutes after the conclusion of the gametocytaemic blood meal, and reached a peak of activity between 5 and 10 minutes post feeding (Figure 1). The earliest invasion of the midgut epithelium by ookinetes was observed 20 hours after blood ingestion. Mature oocysts were found between 8–14 dpi (days post infection), with the earliest oocyst rupture occurring at 9 d post feeding. The earliest sporozoite presence in the salivary gland was observed between day 15 and 16 post feeding (Figure 1), but established salivary gland infections persisted until females mosquitoes can be survived (more than 30 days). It is noteworthy that from all infected insects with *P. vivax*, the overall number of mosquitoes exhibiting sporozoite invasion of the salivary glands was 24% lower than the number supporting oocysts in their midgut (21.9% of the infected mosquitoes exhibited live oocytes on their midguts while 16.6% of the mosquitoes' salivary glands were infected with sporozoites). Together these observations reflect the well-documented bi-phasic population bottlenecks in *Plasmodium*-infected mosquitoes that occur at the critical stages of ookinete invasion of the midgut, and during sporozoite invasion of the salivary glands.

### Carbohydrate inhibitory assays

**Figure 2:**
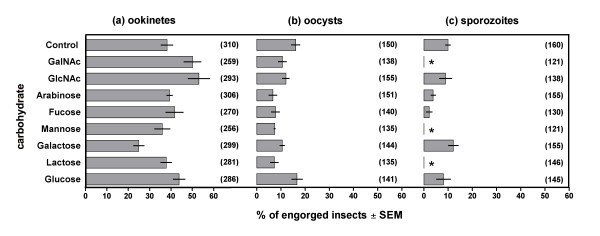
**Inhibitory effect of carbohydrates on the development of *Plasmodium vivax *parasites in *Anopheles stephensi *mysorensis**. The percentage of engorged insects exhibiting **(a) **ookinetes, **(b) **oocysts and **(c) **salivary gland sporozoites is reported as the mean of three independent experiments. For each of the three developmental stages, the number of insects containing parasites in each treatment group was statistically compared with the corresponding control using the Chi Squared test (* P < 0.05). Numbers in parentheses refer to the sample size (n), i.e. total number of engorged insects.

The effects of inhibitory carbohydrates were manifested differently at two critical stages of the *P. vivax *life cycle: (i) ookinete traversal of the epithelial barrier of the midgut and their subsequent development into oocysts, and (ii) sporozoites crossing into the basal membrane of salivary glands. As shown in Figure 2, inhibitor carbohydrates had a much more pronounced effect on sporozoite activities than on ookinetes/oocysts. Only minor perturbations were observed for the development of *P. vivax *oocysts at the basal side of the *An. stephensi *midgut following ingestion of arabinose and fucose with the gametocytemic blood meal, compared with the control group fed without carbohydrates, (Figure 2). No reductions in oocyst development were observed in mosquito groups treated with GlcNAc, GalNAc, galactose, mannose or glucose, compared with the control group.

Strikingly however, sporozoites were completely absent from salivary glands of mosquitoes treated with mannose, GalNAc, and lactose (all P < 0.05 compared with the controls; Figure 2). In contrast, sporozoite development was observed among mosquitoes treated with GlcNAc, arabinose, galactose and glucose at levels similar to those of the controls fed without carbohydrates. A small number of sporozoites were observed in the extruded haemolymph during dissections to remove the intact salivary glands. These were noted following mannose, lactose and GalNAc treatments, as well as the other carbohydrates. This suggests that the inhibitory carbohydrates acted by compromising salivary gland invasion or by intercepting haemoceol sporozoites, and not by exerting effects at earlier stages such as oocyst rupture.

## Discussion

Human malaria is only transmitted via the bite of an infected mosquito, thus blocking its transmission is a potentially effective control measure. It is anticipated that this study will improve our knowledge of the role of carbohydrates in vector-parasite interactions, which can be used for developing transmission-blocking vaccines (TBVs). One important reason why a viable transmission-blocking mechanism is not yet available is because target molecules crucial to the parasite's development in the vector, are not fully understood. Furthermore, the well-known species-specific differences in host-parasite interactions must be considered when developing TBVs targeted to specific geographical regions. From this perspective, the current two-stage study was designed to first examine the competency of *An*. *stephensi *strain mysorensis to support *P. vivax *development (the dominant species of *Plasmodium *in southern Iran), and then to identify specific carbohydrates involved in tissue localization of *P. vivax *in this mosquito host.

The results indicate that parasite development takes 12 days on average to complete its development from exflagellation to sporozoite formation. The average of adult longevity for females of *An. stephensi *is approximately 30 days during the malaria transmission seasons in southern Iran, and each gonotrophic cycle for this vector is 2–3 days [20]. Thus, after 13–14 days this vector can be infectious and transfer the parasites for at least 16 days. Crucially, this means that each female has the potential to survive long enough to be re-infected and thus could transmit *vivax *malaria several times. A major conclusion of the study is that *An. stephensi *mysorensis should be considered a good vector for *P. vivax *in this particular area of Iran.

More urgent research into possible new transmission control strategies is required. This study uncovers new details about the compatibility of *An. stephensi *strain mysorensis with *P. vivax*, the principle vector of vivax malaria in this region of Iran. These crucial data are useful for malaria epidemiology studies in this particular region. In addition, the distribution of this mosquito strain is overlapped by another vector, *An. culicifacies*. It was therefore essential to clarify the role of *An. stephensi *strain mysorensis regarding the transmission of *Plasmodium *in that geographical area, which has a high incidence of malaria infections vectored by insecticide-resistant mosquitoes. It is recognized that differences in the dynamics of host-parasite interactions are strongly dependent on the mosquito/parasite species combination [21], thus, baseline information of *P. vivax *transmission in each area must be identified based on its vectors before planning effective control programs, and then control methods should address local transmission epidemiology.

The initial attachment of vector-borne pathogens to the midgut of their invertebrate hosts involves glycoprotein molecules. In the majority of arthropod vectors, the luminal face of the midgut epithelium is covered by a dense array of glycoconjugates that act as a glycan receptor for a myriad of pathogens [8]. Inhibiting this initial step with conventional methods as well as more novel approaches, such as oligosaccharide mimics or anti-glycan antibodies, could result in a reduction in disease transmission [9]. Clearly, one of the difficulties lies in developing a multivalent inhibitor that can effectively overcome the pathogen adhesion. While this is no easy task, it is likely that glycobiological analyses will make significant contributions toward resolving the mechanisms of attachment of vector-borne pathogens to their invertebrate hosts. It is known that carbohydrates taken up by a mosquito along with a parasite infective blood meal can mask parasite glycan receptors that are critical for attachment. There is evidence to suggest that malaria parasite ookinete ligands have carbohydrate recognition properties [7-22]. It can be envisaged that specific domains of the ookinete envelope glycoprotein are responsible for recognizing different glycans, or mosquito midgut microvillar glycoproteins during initial attachment. Moreover, an antibody against mosquito midgut microvillus epitopes was shown to completely inhibit *Plasmodium yoelii *oocyst development in the midgut of *An. stephensi *[10]. Furthermore, the ability of this antibody to recognize its similar mosquito carbohydrate receptor was significantly inhibited by jacalin (which is specific for Gal moieties on O-glycans) and WGA by a competition enzyme-linked immunosorbent assay [22], suggesting that the antibody shares similar glycan specificities with these two lectins.

The present study demonstrated that sporozoite invasion of the salivary glands was completely inhibited by three carbohydrates: mannose, lactose and GalNAc. Under these conditions, sporozoites were nevertheless observed in the haemolymph, strongly suggesting that the inhibitory activity of the fed carbohydrates was targeted to the salivary gland epithelium and/or the interception of sporozoites *en route *to the epithelium through the haemolymph. Previous studies have detected mannosyl and GlcNAc moieties on mosquito salivary glands by using FITC-labelled lectins, in three strains of *An. gambiae*, two strains of *Anopheles arabiensis *and a single strain of *Anopheles merus *[18]. These data collectively indicate that such sugars block their respective glycan binding proteins, which are used for sporozoite invasion.

It is postulated that sporozoite interaction with the salivary glands is species-specific [19-23] and specific glycosylated proteins might be the receptors on the basal lamina of the distal lateral and the medial lobes of the salivary glands (the regions in which sporozoites are found) [19-24]. Earlier studies also demonstrated that the invasion of *P. gallinaceum *sporozoites into the salivary glands of *A. aegypti *is blocked by a carbohydrate binding protein or lectin, wheat germ agglutinin (WGA), succinyl-WGA39 and antibody raised against this lectin [7], which confirms the role of glycosylated proteins as receptors for interaction of the sporozoite with the mosquito salivary glands. It is beyond the scope of the current study to explain why mannose, lactose and GalNAc so profoundly blocked sporozoite invasion of the salivary glands, yet did not affect midgut invasion. However, it is probable that this was due to developmental stage-specific and tissue-specific differences in host-parasite carbohydrate-receptor interactions. At present, the fields of mosquito and *Plasmodium *glycomics are in their infancy, but it is anticipated that future carbohydrate/oligosaccharide microarrays and other high-throughput glycan analyses will allow a detailed elucidation of stage- and tissue-specific interactions.

Therefore, vector competence is dependent on the contribution of critical glycan, and other protein co-receptors. This recognition mechanism may explain how some anopheline vectors can support mixed plasmodium infections, how some plasmodia can infect multiple anopheles vector species, or how some *Anopheles *are refractory to *Plasmodium *development.

Clearly, the relative contributions of protein or glycan receptors to *Plasmodium *infection of mosquitoes require additional investigation. Glycotypes are potential transmission-blocking vaccine candidates for vector-borne disease and further underline the importance of understanding the effects of carbohydrate epitopes in the development of a mosquito vaccine. TBVs typically target the development of gametocytes or ookinete interactions with the mosquito midgut. The task of blocking invasion of the salivary glands is more challenging because antibodies ingested with the blood meal must first pass through the midgut and haemocoel in a functionally intact form. However, this phenomenon is not without precedent. For example, Okulate *et al *[25] recently described a 73% reduction in sporozoite numbers in salivary glands of *An. gambiae *following ingestion of a monoclonal antibody raised against the salivary gland receptor, saglin. This antibody did not interact with the midgut and was salivary gland specific. It may therefore be possible to successfully develop TBVs based on anti-glycan antibodies, or indeed, antibodies targeting protein receptors for these carbohydrates.

The results presented here indicate that host-parasite sugar binding interactions are essential for development of *P. vivax *in the salivary glands of its vector, and the identification of the sugar binding receptors will assist further studies on TBV development.

## Authors' contributions

HRB conceived, designed and coordinated the study. SD and KA conducted field studies including phlebotomy, infections and mosquito breeding. MN contributed to laboratory work including blood scrrening, and HL performed statistical analyses. HRB, SD and KA performed the experiments. HRB and MMAW wrote, read and approved the final manuscript.
